# Age-Related Risk Factors at the First Stroke Event

**DOI:** 10.3390/jcm9072233

**Published:** 2020-07-14

**Authors:** Raúl Soto-Cámara, Jerónimo J. González-Bernal, Josefa González-Santos, José M. Aguilar-Parra, Rubén Trigueros, Remedios López-Liria

**Affiliations:** 1 Department of Health Sciences, University of Burgos, 09001 Burgos, Spain; rscamara@ubu.es; 2 Department of Psychology, Health Research Centre, University of Almeria, 04120 Almeria, Spain; rtr088@ual.es; 3 Department of Nursing, Physiotherapy and Medicine, Health Research Centre, University of Almería, 04120 Almeria, Spain; rll040@ual.es

**Keywords:** stroke, risk factors, age, elderly

## Abstract

(1) Background: Stroke is a multifactorial disease, which can affect individuals at any age. Risk factors (RFs) associated with the first stroke event have been well identified; however, the influence of these RFs on the patient’s age needs to be studied. (2) Objective: This study aimed to examine the effect of modifiable RFs on the age at which a stroke occurs. (3) Methods: A cross-sectional study was conducted on patients admitted consecutively with a first-ever acute stroke at the Burgos University Hospital (Spain). Data on sociodemographic and clinical parameters were collected (high blood pressure (HBP), smoking habit, diabetes mellitus (DM), dyslipemia, abdominal obesity, sedentary lifestyle, alcohol consumption, and cardiovascular diseases). The possible associations between RFs and age were studied using univariate and multivariate regression analyses and a decision tree. (4) Results: A total of 436 patients with a mean age of 75.39 years (standard deviation (SD) ± 12.67) were included. HBP and overweight/obesity were the most prevalent stroke RFs. Being an active smoker (OR 21.48; 95% confidence interval (CI) 8.80–52.41), having a sedentary lifestyle (OR 3.24; 95% CI 1.97–5.31), being an excessive alcohol drinker (OR 2.36; 95% CI 1.45–3.84), or being overweight or obese (OR 1.95; 95% CI 1.14–3.34) increased the risk of having an acute cerebrovascular event in individuals aged 75 years or below. However, a personal history of HBP (OR 0.40; 95% CI 0.24–0.67) was significantly associated with a greater likelihood of having an acute stroke in individuals aged more than 75 years. (5) Conclusions: This study showed that the modifiable RFs strongly influence the first stroke event in patients aged below 75 years, which will be useful in guiding different prevention strategies.

## 1. Introduction

Stroke is a global public health problem in developed countries, a situation which will be aggravated by the progressive population aging. It is one of the main causes of morbidity and mortality, dependency, and disability. Approximately half of all the patients who survive an acute stroke fail to regain independence and need long-term healthcare, which incurs high costs for the patient, their family, and health services [1,2].

Stroke etiology is multifactorial. Risk factors (RFs) are defined as the confluence of personal, environmental, and social circumstances, which help in identifying a group of people who are more likely to develop a certain disease throughout their lives than the rest of the population [3,4]. Traditionally, stroke RFs have been categorized into two groups on the basis of whether they can be modified: nonmodifiable (sex, age, and race/ethnicity) and modifiable (high blood pressure (HBP), diabetes mellitus (DM), apolipoproteins levels, abdominal obesity, active smoking habit, physical inactivity, alcohol intake, diet, psychosocial factors, and cardiac causes) [3,4]. The INTERSTROKE project, an international multicenter case-control study, has shown the importance of these 10 modifiable RFs in the acute stroke, explaining 90% of the risk. HBP has a greater effect on hemorrhagic strokes whereas current smoking, DM, apolipoproteins, and cardiac causes increase the risk of ischemic ones [5,6].

Stroke can affect individuals of any age, although traditionally it has been perceived as a disease of older people with the incidence rate doubling for each decade after 55 years of age [3,7]. In recent years, the mean age of stroke onset has been decreasing, and stroke incidence and hospitalization rates have been rising globally among young individuals [8,9]. HBP, DM, smoking, low aerobic fitness, and obesity are the prominent stroke RFs in younger patients [9,10]. Modifiable RFs, clinical presentation, and outcomes of stroke differ among patients with different age and sex [11,12,13,14,15]. Younger patients with stroke are at an increased risk of cardiovascular mortality and morbidity compared with the general population [15].

Studies evaluating the younger and older Spanish population after the first stroke are scarce, and whether the RF distribution between both populations is different remains to be elucidated. A better identification and understanding of modifiable stroke RFs in both younger and older patients is necessary to develop and implement effective prevention strategies for reducing the global burden. Therefore, the aim of this study was to examine the effect of modifiable RFs on the age at which a stroke occurs.

## 2. Materials and Methods

This was a cross-sectional descriptive study, which is a part of a broader project researching on prehospital delay and treatment in stroke, whose preliminary results have been published previously [16]. The project was conducted at the Neurological Department of the Burgos University Hospital (Burgos, Spain), a tertiary teaching regional reference center for stroke care, which has an established program for intravenous thrombolysis and a stroke unit.

All patients of both sexes aged more than 18 years admitted consecutively to the emergency department (ED) with the diagnosis of first-ever acute stroke, according to the World Health Organization definition, were candidates for inclusion in the study. Stroke was diagnosed and confirmed by computed tomography and/or magnetic resonance imaging of the brain, performed at the acute stage in all patients. The exclusion criteria for patients included the following: inability to communicate directly because of damaged conscience level or speech, no family member was available to provide the information, and refusal to participate in the study. To avoid bias, if a patient was admitted to the ED because of recurrent strokes during the study period, no event was considered. The data were obtained without any prespecified hypotheses, and the sample size was not previously determined.

Patients and/or next of kin were informed about the purpose of the study and invited to participate. People who were ready to participate in the study were asked to sign the informed consent. All data were collected within 72 h of admission using an ad hoc structured questionnaire that takes 5–10 min in a face-to-face clinical interview. This questionnaire had been previously piloted on a sample of 25 patients. The electronic medical records were reviewed to check or complete information. The research protocol was designed as per the Helsinki Declaration of 1975 and approved by the local institutional review board (Reference 1479).

The main outcome was the age (in years) at which the patient suffered the cerebrovascular event. Other data on sex, HBP, DM, dyslipidemia, overweight/obesity, cardiovascular disease, active smoking, excessive alcohol consumption, and sedentary lifestyle were collected. For the analysis and evaluation of the stroke RFs, the criteria defined by the INTERSTROKE project [5,6] and the Program of Preventive Activities and Health Promotion of the Spanish Society of Family and Community Medicine (PAPPS-SEMFYC) [17,18] were used. Patients were considered to be hypertensive if they had systolic (SBP) or diastolic blood pressure (DBP) of more than 140 mmHg or 90 mmHg, respectively, and/or were taking antihypertensive drugs. DM was defined as a fasting plasma glucose level higher than 7.00 mmol/L, glycosylated hemoglobin higher than 6.5%, and/or taking oral antidiabetic drugs and/or insulin. Dyslipidemia considered one of the following plasma levels: total cholesterol higher than 5.20 mmol/L, high-density lipoprotein cholesterol (HDL-C) less than 1.04 mmol/L, low-density lipoprotein cholesterol higher than 3.40 mmol/L, or triglycerides higher than 1.7 mmol/L and/or taking lipid lowering drugs. Overweight and obesity were defined as a body mass index (BMI) of more than 25 and 30, respectively. Cardiovascular diseases included a previous antecedent of coronary heart disease, arrhythmia, chronic heart failure, and peripheral artery disease. AF was confirmed by electrocardiogram any time before stroke onset or during the hospital stay. A patient was defined as an active smoker if he/she had smoked daily for the past month, regardless of the amount, and/or had stopped smoking in the past one year. Alcohol consumption was recorded excessive if the patient drank more than 9 g of alcohol per day in men and more than 6 g in women. A sedentary lifestyle was defined as performing a physical activity for less than three times per week for at least 30 min each time. The severity of stroke on admission was assessed using the National Institute of Health Stroke Scale (NIHSS) [19].

Absolute frequencies and percentages were used for categorical variables for sample characterization. Age was normally distributed; therefore, for descriptive statistics, the mean values and standard deviation (SD) were calculated. The association between age and RFs was assessed using independent samples Student’s *t*-test. To quantify the magnitude of this association as a whole, the odds ratio (OR) was calculated using a forward stepwise multivariate regression analysis adjusted by sex, which included the variables that were significant in the univariate analysis. Age was dichotomized into two groups using the mean age of stroke onset as the cutoff point, i.e., 75 years. To interpret the results and to understand RFs that influence the age at which an acute stroke occurs, a decision tree was designed on the basis of exhaustive chi-square automatic interaction detection with a K-fold cross validation method. This analysis builds a predictive model to help determine how RFs explain the occurrence of an acute stroke at a specific age. The criteria used to build the decision tree were that the maximum number of levels was 5, the minimum number of cases in parent nodes was 100 and in children nodes 50, the significance level for splitting nodes was 0.05, the significance values were adjusted by Bonferroni method, and the number of sample folds in the cross validation method was 10. A *p* value of < 0.05 was considered statistically significant. Statistical analysis was performed using the SPSS statistical software package, version 25.0 (IBM-Inc., Chicago, IL, USA).

## 3. Results

Of 607 patients admitted to the ED, 171 were excluded for different reasons: a history of stroke before the index event (*n* = 133), refusal to participate in the study (*n* = 24), and inability to communicate data (*n* = 14). The characteristics of this group were similar to 436 patients included in the study with respect to age and sex.

Overall, 55.70% of these patients were male. The mean age was 75.39 (SD ± 12.67) years. Moreover, 19.27% (*n* = 84) of patients were below the age of 65 years, 18.58% (*n* = 81) were between 65 and 74 years, 37.84% (*n* = 165) were between 75 and 84 years, and 24.31% (*n* = 106) were 85 years or above.

In addition, 404 patients presented two or more RFs, having HBP was the most prevalent among them (67.40%), followed by overweight/obesity (64.91%), and cardiovascular disease (49.77%). On the other hand, the least frequent RFs were AF (30.96%), DM (25.00%) and active smoking habit (18.58%). Most of the acute strokes were ischemic, with a mild/moderate severity level (NIHSS ≤ 16) (Table 1).

Women were much older than men when experiencing their first acute stroke (79.07 ± 11.96 versus 72.47 ± 12.48 years; *p* < 0.001). Men were more likely to be active smokers (*p* < 0.001) and to drink excessive alcohol (*p* < 0.001). In contrast, women had a significantly higher prevalence of overweight/obesity (*p* < 0.001) and HBP (*p* < 0.001). No differences were observed between stroke characteristics (type and severity) and sex.

The mean age at which an acute stroke occurred was significantly associated with all the RFs analyzed, except for DM. Increasing prevalence of overweight/obesity (*p* = 0.048), active smoking habit (*p* < 0.001), excessive alcohol consumption (*p* < 0.001) and sedentary lifestyle (*p* < 0.001) with the lowering age at the onset of acute stroke was observed. Patients with HBP (*p* < 0.001), dyslipidemia (*p* < 0.001), cardiovascular disease (*p* < 0.001), or AF (*p* < 0.001) tended to have an acute stroke at a later age (Table 2). However, the mild/moderate severity of the acute stroke (NIHSS ≤ 16) was significantly associated with the younger ages (*p* = 0.002).

In multivariate regression analysis, eight of the nine studied RFs were included (HBP, dyslipidemia, overweight/obesity, cardiovascular disease, AF, active smoking habit, excessive alcohol consumption, and sedentary lifestyle). In this analysis, being an active smoker (OR 21.48; 95% confidence interval (CI) 8.80–52.41), having a sedentary lifestyle (OR 3.24; 95% CI 1.97–5.31), being an excessive alcohol drinker (OR 2.36; 95% CI 1.45–3.84) or being overweight or obesity (OR 1.95; 95% CI 1.14–3.34) increased the risk of having an acute cerebrovascular event in patients aged 75 years or below. However, a personal history of HBP (OR 0.40; 95% CI 0.24–0.67) was significantly associated with a greater likelihood of having an acute stroke in patients more than 75 years (Table 3).

**Figure 1:**
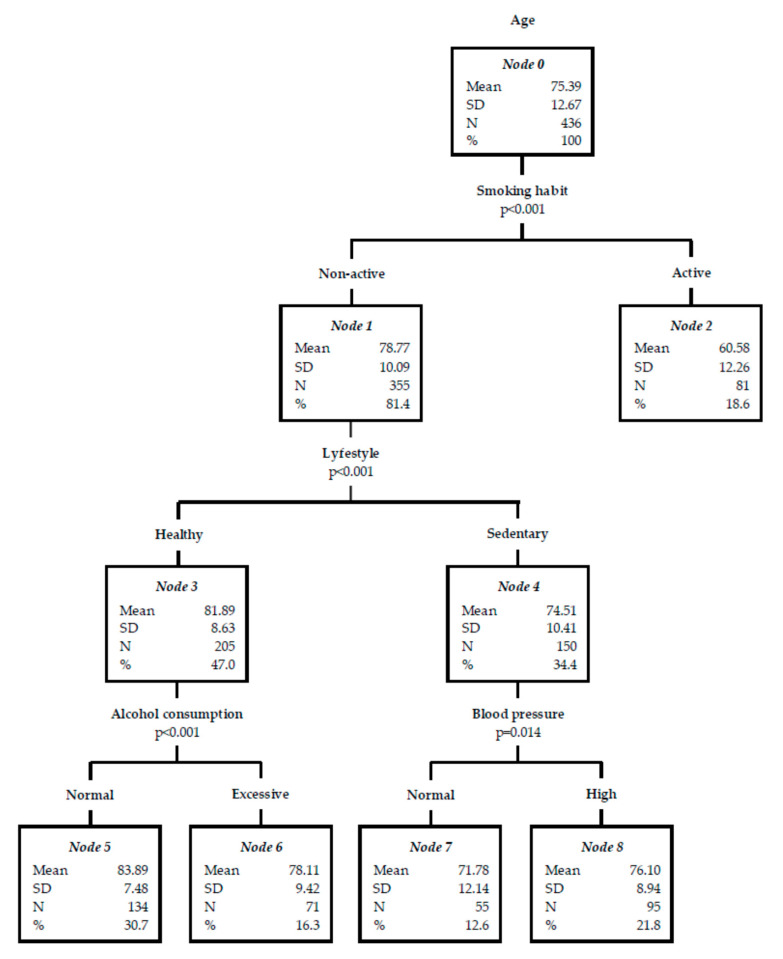
Decision tree for acute stroke risk factors (RFs) based on age.

Finally, to interpret the observed associations and find other specific subgroups and relationships that may not be obtained through a multivariate regression analysis, a decision tree was made (Figure 1). As seen at node 5, no smoking patients, with a healthy lifestyle, and non-excessive alcohol consumption suffered a cerebrovascular event at an older age (mean 83.89 ± SD 7.48 years). On the other hand, according to the results of node 2, a stroke occurs at a lower age among patients with an active smoking habit (mean 60.58 ± SD 12.26 years). In this decision tree, the estimated risk based on the cross validation method was 101.08 (Standard Error = 8.12).

## 4. Discussion

This cross-sectional study has analyzed the relation between different stroke RFs and age. It has demonstrated that being an active smoker, having a sedentary lifestyle, being an excessive alcohol drinker or being overweight or obesity is significantly associated with a greater likelihood of having an acute stroke in patients aged 75 years or below, while a personal history of HBP increase the risk of having an acute cerebrovascular event in patients more than 75 years. These results are in line with other international studies [6,8,11,13,17]. In Spain, the prevalence of the main stroke RFs is high: 50.3% of the subjects have high apolipoproteins levels, 47.4% HBP, 29.7% sedentary lifestyle, 28.2% abdominal obesity, 19% DM, and 18% was an active smoker [20].

Stroke is a disease associated with aging. This nonmodifiable RF increases the incidence of cerebrovascular events, doubling it for each decade after the age of 55 years [7]. Several studies have shown that the established stroke RFs were more common in younger patients than previously assumed [15,21,22]. In this study, several older patients were included; almost three in five patients were above 75 years old. These data reflect the international trend toward the increasing rates of acute stroke among elderly patients [23]. The studied predictive RFs of the first-ever acute stroke have been associated with the patient’s age. Younger stroke survivors had higher proportions of unhealthy lifestyle RFs, such as high BMI, smoking, or excessive alcohol intake, which is consistent with the findings from studies conducted in Israel [24] or Norway [25]. A shorter life expectancy related to the mentioned RFs may explain their lower prevalence among older patients [26]. In accordance with the results of the Framingham study (with 5209 subjects from Massachusetts), cardiovascular diseases, AF, and HBP have been considered crucial RFs in elderly patients with stroke [27].

Globally, stroke onset occurs at a later age in women than men. Moreover, older women have a higher prevalence of DM, cardiac diseases, and atrial fibrillation (AF); whereas men are more likely to drink heavily or smoke than women in a Chinese study [11]. In our study, the distribution of stroke RFs varied in both the sexes, too. The prevalence of HBP and overweight/obesity was higher in women than men; whereas, an active smoking habit or excessive alcohol consumption were fewer in this group, which is consistent with the findings of other studies in China, Germany and Spain [14,28,29,30]. The association between sex and stroke RFs depends on age. Generally, women suffer the first-ever acute stroke at older ages, probably because of the longer life expectancy of women than men [31]. Women live 6–8 years longer than men, which may be because of their biological advantage, mortality differences, lifestyles, and effect of environmental and social factors [32,33].

No traditional RF, other than HBP, was associated with the first-ever acute stroke among those aged more than 75 years. Lifestyle RFs had a stronger association with patients younger than 75 years but not with the oldest ones. Several explanations support these findings; most of them were based on the fact that the associations between lifestyle RFs and stroke were weakened with advancing age [34]. On the one hand, survivors with RFs in the elderly group were more likely to have had events earlier in life, and therefore, survival to older age with RFs may indicate their lower susceptibility to such RFs [34]. On the other hand, the effect of some RFs may be modified because of physiologic changes that occur during the aging process, which are associated with higher vulnerability [35]. In all cases, earlier treatment of RFs can be useful in preventing stroke incidence.

HPB is the most prevalent modifiable RF for stroke, with significant differences between sex and age. The risk of stroke increased with the duration of hypertensive period and age, having a lifetime probability of 90% in those who survived until the age of 80 years [36,37,38]. In people aged 65 years or above, two-thirds were hypertensive; the effect of blood pressure was also greater for hemorrhagic than that for ischemic stroke [3]. In the Suita Study (Japan), the cumulative lifetime stroke risk at the age of 75 years was 11.8% and 13.1% for hypertensive men and women, respectively; this risk lowered to 5.5% and 5.3% for men and women without HBP, respectively [36]. SBP was positively, and DBP was negatively associated with stroke risk with advancing age, whereas the pulse pressure (PP) increased in the elderly. The highest SBP-associated risk occurs in the age group of 60–69 years, declining thereafter. Among patients aged 70–79 years, baseline SBP, DBP, and PP were predictive of stroke in women; however, only SBP and DBP were predictive of stroke in men [39]. Some prevention strategies include identifying and treating medical conditions, such as HBP and DM, which increase stroke risk. Even modest changes in lifestyle are achievable and have a substantial effect on RFs. Modifiable behaviors such as an active smoking habit, inadequate diet, or sedentary lifestyle have a crucial cumulative association with HBP [6]. In our study, HBP was related to the female sex and older age when an acute stroke occurs, which is in line with the results of other studies in Japan, USA, Sweden, or Norway [13,39,40,41]. A possible explanation for these findings may be that both primary and secondary prevention measures were likely to affect the rate of stroke in older patients [42]. However, some studies have concluded that HBP was not influenced by sex or age [11]. This difference in study results may be because of different racial and environmental factors in the pathogenesis of HBP.

Physical inactivity is associated with several poor health effects, including stroke. In this study, patients who do not exercise regularly were three times more likely to have a stroke at younger ages. A sedentary lifestyle is itself an RF for most metabolic diseases (HBP, DM, dyslipidemia, and overweight/obesity) and is correlated with other unhealthy behaviors (active smoking habit and excessive alcohol consumption) [12]. Low physical activity has been identified as an RF for stroke in young patients, both men (46.6%) and women (50.4%) in another study [22]. A meta-analysis on physical activity and stroke risk showed that moderately and highly active adults have a lower risk of stroke than those having low activity. Moderately and highly active adults have a 20% and 27% lower stroke risk, respectively [43]. Physical activity has several benefits such as lowering blood pressure and adiposity; improving HDL-C and triglyceride levels; reducing blood viscosity, platelet aggregability, and fibrinogen levels, enhancing endothelial function; reducing stress levels; improving mood; and facilitating engagement with healthy behaviors [44,45]. Physical activity may reduce the risk of DM [3]. Despite the benefits of an active life, a sedentary lifestyle continues to be an increasing trend. Preventive programs to reduce the incidence of stroke in younger people should include a schedule of balanced physical activities.

For overweight or obese patients, the probability of having a first acute stroke was two times higher in the younger population. Overweight and obesity significantly increase cardiovascular risks in general and the risk of stroke specifically, particularly in younger people [46,47]. Obesity increases stroke risks because of its association with prothrombotic and proinflammatory states or other severe RFs related to vascular diseases such as HBP, DM, or dyslipidemia. Several studies have shown the independent association between BMI and the risk of stroke, although the results have been contradictory in several aspects [3,4,48]. In addition to BMI, a central obesity pattern, characterized by the abdominal fat deposits and defined as a waist-hip ratio of more than 102 cm in men and more than 88 cm in women, has been strongly associated with an increased risk (up to three times) of ischemic stroke, particularly in men [4,7,48]. As the prevalence of childhood and young adulthood obesity has increased in the past years, innovative and effective screening programs are urgently needed to work on this modifiable factor for reducing the stroke risks.

Active smoking habit is a major RF that influences the age at which an acute stroke occurs, being 20 times more likely in younger people; these results were in line with those obtained by other studies in USA [49,50]. Although this RF was more prevalent in younger men, several studies have revealed two to three times higher risk of hemorrhagic stroke in younger women who smoke [51]. A strong dose–response relationship exists, i.e., the stroke risk increases with the increasing number of cigarettes smoked per day. Smoking cessation rapidly reduces the stroke risk, decreasing proportionately with the time elapsed and being equal to the risk of the nonsmokers at five years [49,50,52]. Smoking leads to atherosclerosis progression; increases hematocrit, platelet aggregation, and fibrinogen plasma levels; decreases HDL-C plasma levels; and increases blood pressure. In addition, smoking has a synergistic effect with other RFs such as HBP, DM, sedentary lifestyle, or contraceptive use [53]. Passive smokers have an increased stroke risk, as this may lead to atherosclerosis progression [54]. Thus, an active smoking habit is one of the most appropriate modifiable RFs for the primary prevention of stroke in a young population.

Excessive alcohol intake is associated with a higher likelihood of having an acute stroke in patients aged less than 75 years, as shown by other study completed in 32 countries (Asia, America, Europe, Australia, the Middle East, and Africa) [6]. A J-shaped relationship exists between alcohol consumption and stroke risk [55,56]. Light or moderate alcohol consumption (≤2 drinks per day in men and ≤1 drink per day in women), as a part of a healthy lifestyle, was a protective factor against stroke; whereas, excessive drinking was associated with an increased stroke risk [56,57]. Several studies have found regional differences in the association between alcohol consumption and stroke or other cardiovascular diseases, explained by differences in drinking pattern and type of alcohol. Excessive alcohol consumption was associated with increased HDL-C levels; decreased platelet aggregation and fibrinogen plasma levels; induced alterations in coagulation factors; and increased risk of HBP, AF, cardiomyopathy, and DM, which individually and synergically predispose to ischemic stroke [58,59]. As the initiation of alcohol consumption at an early age can have long-term consequences in life, corresponding preventive and educative programs should be considered for children and youth.

In this study, stroke RFs are distributed similarly to other studies, with some differences, which may be due to the characteristics of the sample analyzed or cultural components [6,17,20]. The IBERICAN project, a longitudinal study focused on cardiovascular RFs in Spanish adults treated in primary health care, has shown that its prevalence is increasing in the last years due to its low control [20]. These results indicate that adults should regularly visit a physician to check over their health and stroke or heart disease RFs [7]. Therefore, a better understanding of modifiable stroke RFs in younger and older patients is necessary to develop and implement effective preventive strategies for reducing the global burden. Primary care or public health campaigns should focus their efforts, for example, in increasing physical activity in the younger population to encourage smoking and drinking cessation or having a healthy diet and weight control.

This study had several limitations. First, only the eight most common stroke RFs were selected, although these may interact with cultural aspects or potentiate one another, causing recurrent stroke as psychosocial factor, stress or depression, or diet risk score. Hypertension could be divided into stage 1 (140–160 systolic) and stage 2 (>160 systolic); or the age group thresholds could be dichotomized (e.g., 55 years of age) as in previous research. Second, this study was conducted in a tertiary care referral center for neurological disease (unicentric study), with a small sample, which makes it difficult to generalize the results. The generalization to other settings may be supported as participant characteristics were quite similar to those reported in several previous longitudinal studies in terms of age, sex, or symptoms. All these studies have used an epidemiological approach to understand the role of RFs in stroke. The strength of this study was the exhaustive data collection and the method used for collecting these data.

## 5. Conclusions

This study showed the influence of different modifiable RFs on the age at which a stroke occurs. Being an active smoker, drinking excessive alcohol, not doing physical activity, or being overweight or obese, all of which are lifestyle RFs, were significantly associated with a higher likelihood of having an acute stroke in patients aged less than 75 years. However, in older people aged more than 75 years, the most prevalent RF was having a personal history of HBP. The knowledge of stroke risk profiles at every stage of life or age may be useful for supporting different prevention strategies, including identification and resolution of medical conditions, particularly those with environmental interactions that increase stroke thresholds of risk.

## Figures and Tables

**Table 1:** Characteristics of patients.

Risk Factor	*n* (%)
Sex: Male	243 (55.73)
Age-X (SD)	75.39 (±12.67)
High Blood Pressure	294 (67.43)
Diabetes Mellitus	109 (25.00)
Dyslipidemia	216 (49.54)
Overweight/Obesity	283 (64.91)
Cardiovascular Disease	217 (49.77)
Atrial Fibrillation	135 (30.96)
Active Smoking Habit	81 (18.58)
Excessive Alcohol Consumption	199 (45.64)
Sedentary Lifestyle	202 (46.33)
More than Two Risk Factors	404 (92.66)
Type of Stroke: Ischemic	373 (85.55)
Basal NIHSS Score: ≤16	368 (84.40)

*n*—number of patients; X—mean; SD—standard deviation; NIHSS—National Institute of Health Stroke Scale.

**Table 2:** Comparison of the presence of acute stroke risk factors (RFs) based on age, using independent samples Student’s *t*-test.

Risk Factor	Yes		No		*p* Value
	*n*	Age, Years X (SD)	*n*	Age, Years X (SD)	
High Blood Pressure	294	78.36 (10.10)	142	69.25 (15.08)	<0.001
Diabetes Mellitus	109	76.00 (10.55)	294	75.19 (13.31)	0.518
Dyslipidemia	216	77.66 (10.29)	220	73.16 (14.32)	<0.001
Overweight/Obesity	283	74.51 (11.73)	153	77.03 (14.14)	0.048
Cardiovascular Disease	217	79.03 (9.82)	219	71.79 (14.09)	<0.001
Atrial Fibrillation	125	79.84 (8.85)	301	73.40 (13.60)	<0.001
Active Smoking Habit	81	60.58 (12.26)	355	78.77 (10.09)	<0.001
Excessive Alcohol Consumption	199	70.63 (12.68)	237	79.39 (11.23)	<0.001
Sedentary Lifestyle	202	70.75 (12.75)	234	79.40 (11.17)	<0.001

*n*—number of patients; X—mean; SD—standard deviation.

**Table 3:** Multivariate regression analysis of predictive risk factors (RFs) for having an acute stroke in patients aged 75 years or below.

Risk Factor	Odds Ratio	95% Confidence Interval	*p* Value
High Blood Pressure: Yes	0.40	0.24–0.67	<0.001
Overweight/Obesity: Yes	1.95	1.14–3.34	0.015
Active Smoking: Yes	21.48	8.80–52.41	<0.001
Excessive Alcohol Consumption: Yes	2.36	1.45–3.84	0.001
Sedentary Lifestyle: Yes	3.24	1.97–5.31	<0.001
